# Sleep impairment and altered pattern of circadian biomarkers during a long-term Antarctic summer camp

**DOI:** 10.1038/s41598-023-42910-8

**Published:** 2023-09-25

**Authors:** Michele Macedo Moraes, Alice Lamounier Marques, Leandro Borges, Elaine Hatanaka, Debora Heller, Cristian Núñez-Espinosa, Dawit Albieiro Pinheiro Gonçalves, Danusa Dias Soares, Samuel Penna Wanner, Thiago Teixeira Mendes, Rosa Maria Esteves Arantes

**Affiliations:** 1 Department of Pathology, Institute of Biological Sciences, Universidade Federal de Minas Gerais, Belo Horizonte, MG Brazil; 2 Center for Newborn Screening and Genetics Diagnosis, Faculty of Medicine, Universidade Federal de Minas Gerais (NUPAD-FM/UFMG), Belo Horizonte, MG Brazil; 3 Post-Graduation Program in Social Sciences in Development, Culture and Society, Universidade Federal Rural do Rio de Janeiro, Seropédica, RJ Brazil; 4 Interdisciplinary Program in Health Sciences, Universidade Cruzeiro do Sul, São Paulo, SP Brazil; 5 Hospital Israelita Albert Einstein, São Paulo, SP Brazil; 6 Post-Graduate Studies in Dentistry, Universidade Cruzeiro Do Sul, São Paulo, SP Brazil; 7 Department of Periodontology, School of Dentistry, UT Health San Antonio, San Antonio, TX USA; 8 Escuela de Medicina, Universidad de Magallanes, Punta Arenas, Chile; 9 Centro Asistencial Docente y de Investigación, Universidad de Magallanes, Punta Arenas, Chile; 10 Interuniversity Center for Healthy Aging, Chilecito, Chile; 11 Exercise Physiology Laboratory, School of Physical Education, Physiotherapy and Occupational Therapy, Universidade Federal de Minas Gerais, Belo Horizonte, MG Brazil; 12 Department of Physical Education, Faculty of Education, Universidade Federal da Bahia, Salvador, BA Brazil

**Keywords:** Physiology, Medical research, Human behaviour

## Abstract

Antarctic expeditions include isolation and exposure to cold and extreme photoperiods (with continuous natural light during summer) that may influence psychophysiological responses modulated by luminosity and sleep. We assessed changes in night sleep patterns by actigraphy, salivary biomarkers, and perceptual variables in seven participants in the following time points along a 50-day camping expedition in Antarctica (Nelson Island): Pre-Field (i.e., on the ship before camp), Field-1, Field-2, Field-3, Field-4 (from 1st to 10th, 11th to 20th, 21st to 35th and 36th to 50th days in camp, respectively), and Post-Field (on the ship after camp). We also characterized mood states, daytime sleepiness, and sleep quality by questionnaires. Staying in an Antarctic camp reduced sleep efficiency (5.2%) and increased the number of awakenings and wakefulness after sleep onset (51.8% and 67.1%, respectively). Furthermore, transient increases in time in bed (16.5%) and sleep onset latency (4.8 ± 4.0 min, from Pre- to Field-3) was observed. These changes were accompanied by an altered pattern of the emerging circadian marker β-Arrestin-1 and a trend to reduce nocturnal melatonin [57.1%; *P* = 0.066, with large effect size (*ES*) from Pre-Field to Field-2 (*ES* = 1.2) and Field-3 (*ES* = 1.2)]. All changes returned to Pre-Field values during the Post-Field. The volunteers reported sleep-related physical complaints (feeling of cold and pain, discomfort to breathe, and cough or loud snoring), excessive daytime sleepiness, and reduced vigor during the camp. Thus, a 50-day camp alters neuroendocrine regulation and induces physical discomfort, which may explain the impaired sleep pattern and the consequent daytime sleepiness and mood changes.

## Introduction

Antarctic expeditions include psychophysiological challenges due to isolation and exposure to cold and extreme photoperiods—the ICE conditions^1–7^. Photoperiods in polar environments differ from other Earth environments because of the constant or near-to-constant sunlight in summer and darkness in winter. The extreme photoperiods are determinant for Antarctica to be considered one of the harshest places to live and work on our planet^7^. For example, in summer camps, the expeditioners^8^ usually conduct outdoor fieldwork throughout the day, exposed to a light incidence of 40,000 lx^7^ corresponding to direct sunlight. Furthermore, during the night, the only barrier against sunlight is the tent's canvas, which does not entirely block the natural light.

Ambient light is the key *zeitgeber* (i.e., *zeit* "time" and *geber* "giver") of the body pacemaker, localized in the suprachiasmatic nucleus (SCN) of the hypothalamus^9,10^. Thus, the light summer regimen in Antarctica possibly influences endocrine system-related circadian rhythms once darkness stimulates melatonin secretion (which signals the beginning of the nocturnal period)^11^, and exposure to light and stressful conditions stimulates cortisol secretion^12–14^. Thus, it is hypothesized that prolonged daily exposure to sunlight could alter the secretion of hormones (e.g., reduce melatonin and increase cortisol) and change the organism's circadian rhythm regulation.

Hormonal changes related to luminosity have already been observed in the Antarctic field^5,7^, including the phase-delayed secretion of melatonin linked to light exposure until bedtime^7^. As melatonin acts as non-photic information for SCN, this delay can impair sleep induction^9^. Therefore, the polar light regimen may affect the sleep–wake cycle, resulting in the so-called "Polar insomnia" that makes it challenging to fall and stay asleep^15^. As SCN controls peripheral clocks, circadian biomarkers (measured in body fluids) reflect the internal body clock of individuals. β-Arrestin 1 (ARRB1) is a protein that emerged as a chrono-biomarker that is detectable in saliva and expressed in a circadian manner^16^. Therefore, this chrono-biomarker may help understand circadian and sleep changes to the extreme light regime during the austral summer.

Maintaining an optimal thermal environment supports high-quality sleep^17^. However, staying in Antarctica implies exposure to low temperatures, strong winds, storms, and white-out situations, since individuals sleep in tents without heating. Thus, unfavorable thermal conditions can be an additional factor influencing the sleep pattern of the expeditioners once a cold environment reduces skin temperature and increases wakefulness and sleep latency^18,19^.

Pattyn et al.^7^ assessed the sleep pattern of nine individuals over one night by polysomnography after three weeks of sleeping in tents during an Antarctic camp in the summer. They showed decreased sleep efficiency (SE) and sleep latency (SOL), and increased waking after sleep onset (WASO). In addition, changes in sleep architecture were observed, with the delayed onset of slow-wave sleep and the advanced occurrence of the R stage. Although it allows access to sleep architecture, polysomnography is not feasible to monitor and describe the sleep pattern over the entire period due to difficulties in collecting data in an Antarctic field, as also highlighted by Pattyn et al.^7^. An alternative method to address the wake-sleep cycle over time is actigraphy, registered by a wristwatch-like device. Weymouth and Steel^20^ used actigraphy to obtain data during an Antarctic camp in the Scott Base region and did not observe sleep disturbances or changes in sleeping hours. However, these authors followed the participants for just seven nights, which may have been insufficient time to visualize any effect of ICE conditions on sleep patterns.

The reduced sleep quality leads to milder disturbances (i.e., impairments in initiating or maintaining sleep or predominance of nonrestorative sleep)^21^, is associated with daytime sleepiness, contributes to adverse mood changes^22–25^, and precedes the surging of depression^26^. Camps also impose isolation, including communication restrictions. It is worth noting that isolation can induce mood alterations, which, in turn, may influence sleep quality and quantity^27^; therefore, there is a reciprocal relationship between these sleep characteristics and mood changes. In addition, sleep and autonomic nervous activity are interdependent^19^. Thus, fragmented and interrupted sleep may induce stress-related responses, affecting cardiovascular autonomic regulation^28^ and augmenting heart rate.

Despite the extreme daylight pattern faced during an Antarctic summer camp, the possible changes imposed by these conditions and the time course of modifications are unclear. Most studies on sleep disorders in Antarctica collected data on stations, where individuals sleep in well-sheltered places and, in some cases, combined with exposure to high altitudes (above 2,800 m) [for review, see ^27^. Thus, it is necessary to understand the sleep pattern over time during Antarctic summer in camping situations. This is of utmost importance to stablish interventions to improve the sleep quality and, consequently, health, well-being, and safety of isolated individuals without (or restricted in) communication for weeks to months.

In the present study, we assessed activity-sleep patterns by daily actigraphy and also measured biomarkers of circadian rhythm [β-Arrestin 1 (ARRB1), melatonin, and cortisol] in saliva during a seven-week camp in the austral summer (December-January) in Antarctica (South Shetland Island), a condition with maximal exposure to constant natural luminosity. Also, we investigated the relationship between the sleep–wake cycle and daytime sleepiness, sleep quality, and mood states.

## Methods

### Ethics

The present study, which was approved by the Research Ethics Committee of the Universidade Federal de Minas Gerais (19092819.8.0000.5149/ 3.744.162), followed the regulations established by the Brazilian National Health Council (resolution 466/2012) and the Declaration of Helsinki on ethical principles in human beings. The volunteers were informed about the research objectives and the experimental procedures before giving their written informed consent to participate in this study.

### Subjects and experimental approach to the problem

Seven (five men and two women) non-military Brazilian individuals [age: 32.3 ± 8.4 y, body mass: 69.8 ± 16.2 kg, ∑skinfold (triceps, subscapular, pectoral, mid-axilla, abdominal, supra iliac, and mid-thigh): 128.0 ± 49.2 mm, body fat: 20.1 ± 7.4%] were recruited to participate in this study, which followed a descriptive longitudinal approach. The participants took part in ship travel to Antarctica, followed by prospective fieldwork. The entire expedition lasted 62 days: seven days on board Brazil's Navy polar ship "Ary Rongel" (number of tack H-44), 50 days (49 nights) living in a camp settled in Nelson Island located in the South Shetland Islands (S 53.178533°/O 70.899750°), and five days on Brazil's Navy polar ship "Almirante Maximiano" (number of tack H-41). The expedition was divided into six different time points as follows: Pre-Field (i.e., seven days on the ship before camp), Field-1, Field-2, Field-3, Field-4 (from 1st to 10th, 11th to 20th, 21st to 35th and 36th to 50th days in camp, respectively), and Post-Field (five days on the ship after camp). The volunteers' sleep pattern was assessed throughout the expedition by daily actigraphy. The collection of saliva samples and the application of questionnaires occurred at the time points mentioned in the next paragraph. Our experiment was conducted between December 2019 and February 2020 during the Antarctic summer season.

**Figure 1:**
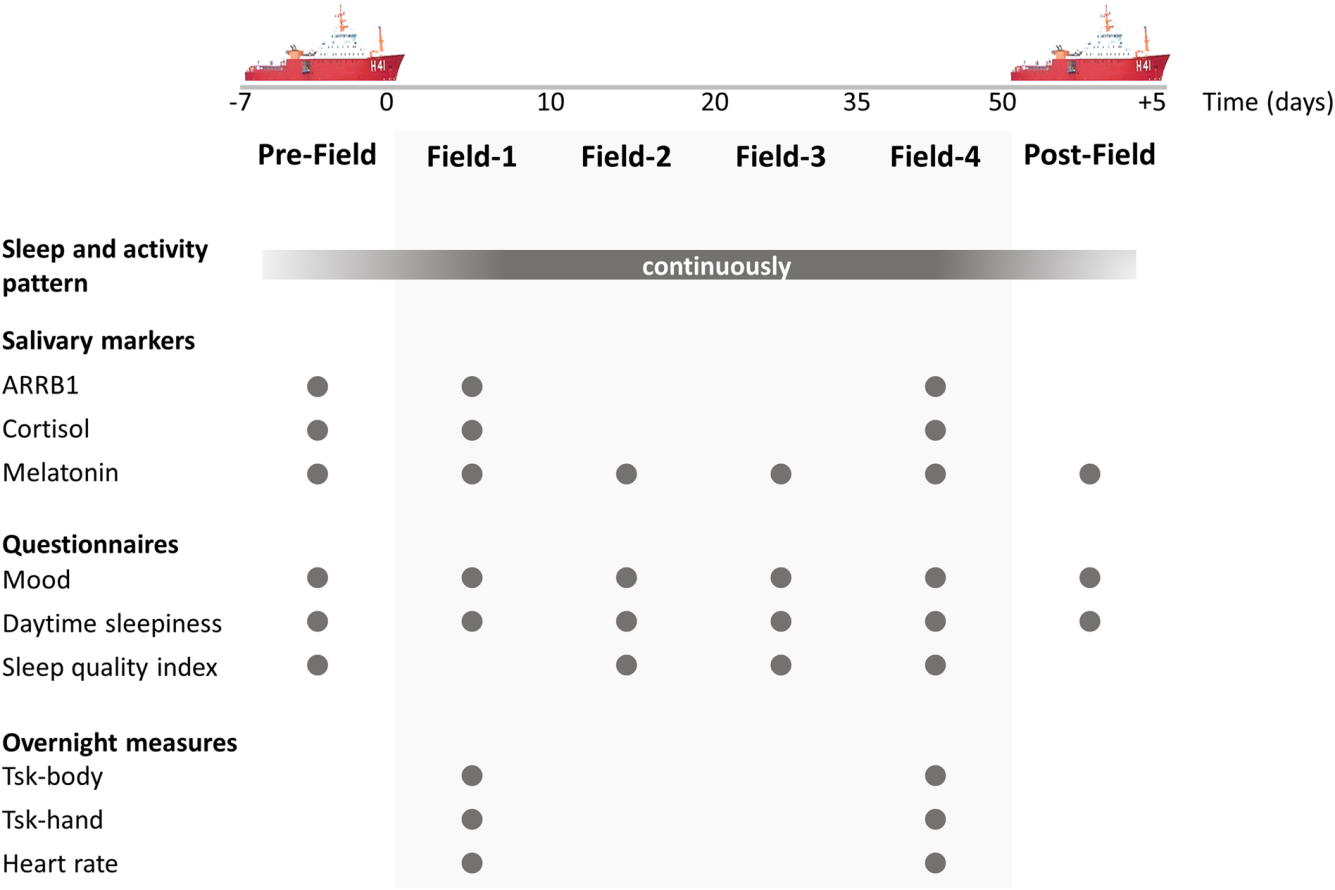
Experimental design. Data were collected in the following time points: Pre-Field (on the ship), Field-1, Field-2, Field-3, Field-4, and Post-Field (on the ship). The gray circles represent the measurements made at each point. Abbreviations: β-Arrestin 1 (ARRB1); skin temperature (Tsk).

The collection of sleep pattern data was done from 24 h after boarding the ship (Pre-Field) until the 5th day after returning to the vessel at the end of the expedition (Post-Field) and included the entire camp period. Saliva samples and application of questionnaires were conducted at the following time points: Pre-Field (2nd and 3rd days), Field-1, Field-2, Field-3, Field-4 4th, 19th, 33rd, and 45th days in camp, respectively), and Post-Field (4th day on the ship) (Fig. 1). The first data collection took place in the Antarctic maritime. The last data collection took place on the 4th day on the ship to avoid data collection during the ship's passage through the turbulent waters of the Drake Passage. The ship started the trip back to Chile through Drake's Passage on the night after dismantling the camp (i.e., the first day on board); this crossing lasted approximately 40 h.

**Figure 2:**
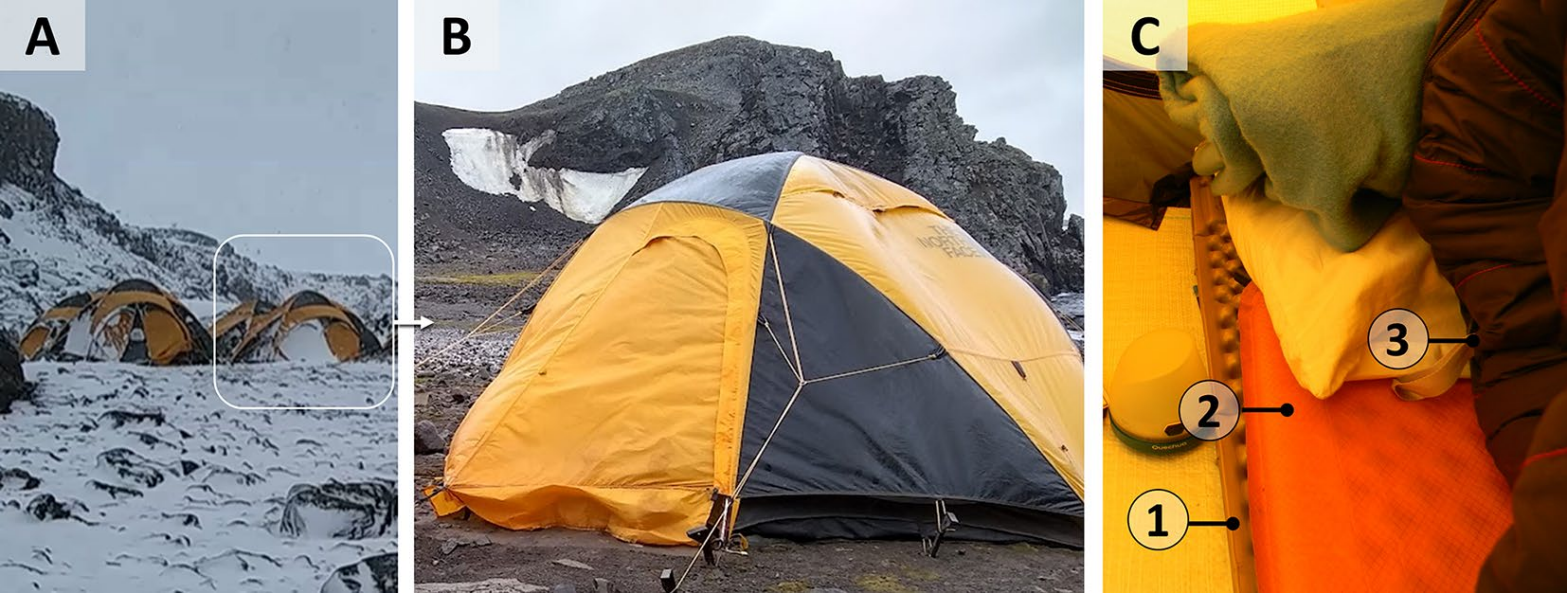
Images of the camping tents. (**A**) View of individual tents. (**B**) A closer view of an individual tent. (**C**) Internal view of a tent, showing the sleeping material: 1. polyethylene thermal insulator of egg-crate type, 2. air-inflatable mattress, 3. sleeping bag.

Concerning the field expedition, the volunteers spent their first days assembling the structures of the camping site, and the first data collection was performed on the 4th day. Tents and sleeping materials were similar for all volunteers. During camps, individuals slept in individual tents (Summit series™ VE25, North Face), inside 'cocoon' sleeping bags (for negative temperatures, -18 °C) propped up on air-inflatable mattresses. These mattresses were insulated from the floor by a polyethylene thermal insulator of egg-crate type. Tents used in camps protect the individuals from the external environment, but external light can still pass through the fabric layer (Fig. 2).

The volunteers followed the same experimental protocol during data collection on the ship and at camp. Saliva samples were collected during fasting state in the morning upon waking (between 6:00–8:00 h; for cortisol measurements), 30 min after waking up (i.e., 6:30–8:30 h; cortisol, melatonin, and ARRB1), at 13:00 h (ARRB1), 19:00 h (cortisol and ARRB1), and 22:30 h (melatonin and ARRB1), with the volunteers seated. The volunteers also answered the questionnaires in the morning, between 06:30 and 09:00 h. Characterization of a typical day on the ship and at camp is presented in Supplemental Material 1 (Box S1).

To understand if body temperatures would influence the night's sleep and characterize the possible effects of sleep patterns on autonomic variables, we evaluated the skin temperatures (Tsk; measured on the hand, arm, chest, and thigh) and heart rate (HR) responses throughout the evening in two moments during the Antarctic field: at the beginning (Field-1, from 4 to 11th day) and end (Field-4, from 38 to 45th day) of the camp (more details and data presented in Supplemental Material 2). The recording was started between 18:00 h and 19:00 h, and the volunteers wore the sensors throughout the night and subsequent day.

### Procedures

#### Sleep and activity patterns measured by actigraphy

Continuous nondominant armband actigraphy was recorded for 24 h at 1-min intervals using wrist-worn activity watches (ActTrust, Condor Instruments, SP, Brazil). Data were analyzed using the Condor software with an algorithm based on the Cole-Kripke method^29^. The wristwatch activity data were extracted in PIM Mode (Proportional Integrating Measure), a measure of activity level or vigor of motion. Sensors of the armband actigraphy recorded wrist temperature and light exposure. The data were analyzed with the Act Studio software (Condor Instruments, São Paulo, Brazil).

The following sleep parameters were evaluated: time in bed (min; time elapsed from going to bed until getting out of bed), sleep onset latency (SOL, min; time in bed until sleep onset), total sleep time (h; the actual time sleeping), sleep efficiency (%; ratio between total sleep time and time in bed), wakefulness after sleep onset (WASO, h; total amount of wake time after the first episode of sleep), fragmentation (number of wake events per night), sleep onset (time of the day of sleep onset), and sleep offset (time of the day of sleep offset). Sleep diary records provided additional helpful information adjunct to actigraphy for editing data to remove artifacts. Also, the volunteers were instructed to use the actigraph button 'event marker' to mark the time they would go to bed and the time they woke up, thus contributing to identifying the onset of sleep and time in bed. Light intensity measured by the actigraphy and the magnitude of the activity recorded also aided in identifying the time in bed accurately. The participants' activity pattern, wrist temperature, and luminosity parameters were also evaluated using the wristwatch (Supplemental Material 2, Table 2).

### Salivary analysis

Unstimulated whole saliva samples (1.5-mL) were obtained by passive drooling (salivation directly into the collection tube) at each collection point to determine the concentrations of melatonin and ARRB1. ARRB1 is a protein that emerged as a chrono-biomarker detectable in saliva and expressed in a circadian manner^16^. In addition, another 0.5-mL unstimulated whole saliva sample was obtained to determine cortisol concentration.

The saliva samples collected on the ship were stored in a − 80 °C freezer. In the Antarctic field, the 1.5-mL samples were stored in liquid nitrogen, and the 0.5-mL samples were stored inside a box buried in ice. After 50 days in the camp, the samples were transferred to the − 80 °C freezer inside the ship and kept frozen until processing and analysis. Before salivary analysis, the mucins and precipitants were removed by centrifugation (14,000 g for 20 min at 4 °C; Eppendorf, 5430 R). Ten percent of the samples were assessed in duplicate to calculate the intra-assay coefficient of variance (CV). Cortisol, melatonin, and ARRB1 were measured in the whole saliva supernatant, according to the manufacturer's protocols, using the following kits: Cortisol ELISA [Salimetrics (State College, PA, USA), dilution 1:5, CV = 3%], Salivary Melatonin ELISA (Salimetrics, dilution 1:3, CV = 5%], and Human ARRB1 ELISA Kit [Wuhan Fine Biotech Co., Ltd (Hubei, China) dilution 1:2, CV = 9%]. Each well's optical density was immediately assessed with a microplate reader set to 450 and 540 nm (wavelength correction). The final values were normalized to the total salivary protein (Pierce™ BCA Protein Assay Kit, ThermoFisher Scientific, Waltham, USA). For ARRB1, the peak value on each collection day (for each volunteer) was set to 1 (100%), and the other measurements of the same day were scaled to the peak value; restricted cubic splines were applied to model a smooth curve mean profile. Melatonin and cortisol concentrations were normalized to Pre-Field values for each volunteer (i.e., value/pre-field value).

### Mood responses, daytime sleepiness, and sleep quality questionnaires

Mood responses were assessed using the 24-item Brunel Mood Scale (BRUMS)^30,31^. Self-reported level of daytime sleepiness was assessed using the Epworth Sleep Scale (ESS)^32,33^. The sleep quality index was evaluated using the Pittsburgh Sleep Quality Index (PSQI)^34,35^, adapted to assess sleep quality over one-week intervals^35–37^.

### Statistical analyses

The Shapiro–Wilk test revealed that all the parameters evaluated did not significantly shift from the normal distribution, except melatonin at 22:30 h. The equal variance was tested and confirmed using the Levène Median test. Data are shown as means ± standard deviations (SD).

One-way repeated-measures (RM) analyses of variance (ANOVAs) were used to compare sleep parameters, sleep quality, salivary biomarkers, daytime sleepiness, mood, activity pattern, wrist temperature, and HR across time points during the expedition. A one-way ANOVA on Ranks (Friedman test) was used to compare melatonin at 22:30 h across time points. Two-way RM ANOVAs were used to compare temperature parameters between different (times of day and expedition time points). When a significant *F* value was found, we performed Duncan (for quantitative sleep and activity parameters by actigraphy, sleep quality, and mood scores) or Student–Newman–Keuls (for hormones, HR, and temperatures) tests as post hoc analyses. Paired Student's *t*-tests were used to compare two means, such as HR and luminosity responses, between the beginning and end of the camp. Pearson’s correlation was used to evaluate the association between two variables by determining the r coefficient. Spearman's correlation was used to assess ranked values within a dataset. The α level was set at 0.05. All the analyses mentioned earlier were performed using the SigmaPlot 11.0 software (Systat Software Inc., San Jose, CA, USA).

Due to the limited number of subjects joining the expedition (n = 7), we also calculated the Cohen's d effect size (*ES*) as a supplementary analysis to understand our findings. Cohen's d was calculated by subtracting the mean value of a time point from the mean value of another time point to which it was being compared. The result was then divided by a combined SD of the data. The *ES* for ANOVAs was calculated using the equation *η2* = Effect SQ / Total SQ; where SQ = sum of squares. The η2 values were converted into *d* values^38^. For non-parametric analyses, *ES* was calculated from the z values of the Wilcoxon signed-rank test. The ES values were classified as trivial (< 0.2), small (0.2–0.6), medium (0.6–1.2), or large (≥ 1.2)^39^.

## Results

### Sleep pattern

**Figure 3:**
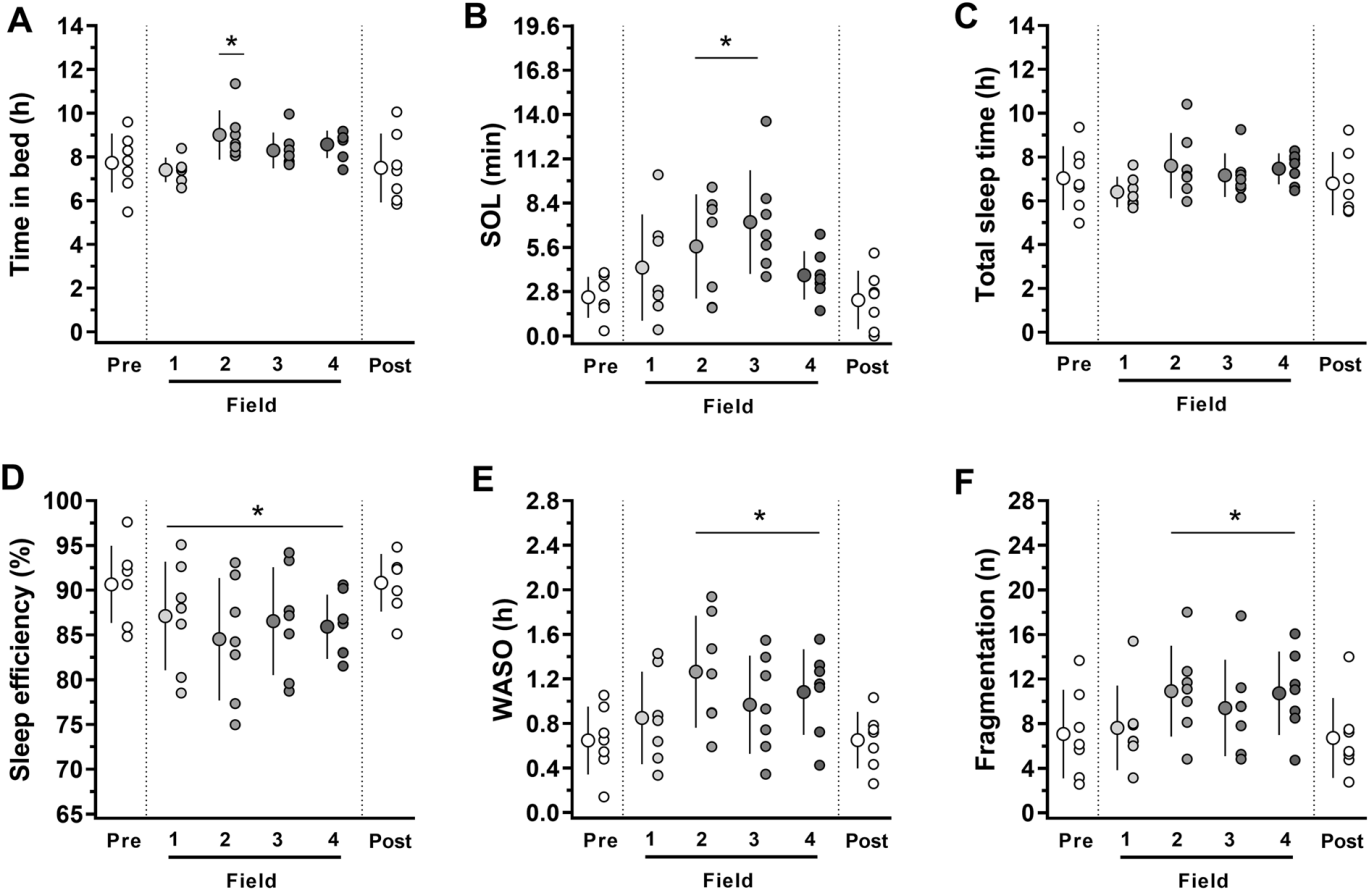
Sleep parameters at Pre-Field (i.e., from 1st to 7th day on the ship), Field-1, Field-2, Field-3, Field-4 (i.e., from 1st to 10th, 11th to 20th, 21st to 35th and 36th to 50th days in camp, respectively) and at Post-Field (i.e., from 2nd day to 5th day on the ship): (**A**) time in bed (min; time elapsed from going to bed time until getting out of bed), (**B**) sleep onset latency (SOL, min; time in bed until sleep onset), (**C**) total sleep time (h; actual time sleeping), (**D**) sleep efficiency (%; ratio between total sleep time and time in bed), (**E**) wake after sleep onset (WASO, h; total amount of wake time after the first episode of sleep), (**F**) fragmentation (number of wake events per night). The data are expressed as means ± SD. The dots represent individual data. *Significantly different (*P* < 0.05) from Pre- and Post-Field. n = 7.

The one-way RM ANOVAs revealed that the Antarctic camp influenced time in bed (F = 3.31, *P* = 0.017; *ES* = 1.1) (Fig. 3A) and SOL (F = 4.170, *P* = 0.005; *ES* = 1.4), with the post hoc analysis revealing an increase in SOL at Field-2 and Field-3 (Fig. 3B). In contrast, no changes in the total sleep time were observed (F = 2.06, *P* = 0.10; *ES* = 0.7) (Fig. 3C). Moreover, Antarctic camp decreased sleep efficiency (F = 7.33, *P* < 0.001; *ES* = 1.0) (Fig. 3D) for all time-points in camp compared to pre-and post-camp, and increased WASO (F = 7.14, *P* < 0.001; *ES* = 1.2) (Fig. 3E) and number of arousals, i.e., sleep fragmentation (F = 6.55, *P* < 0.001; *ES* = 0.9) (Fig. 3F). The decrease in sleep efficiency and increase in fragmentation with camping can be visualized in the daily data presented in Supplemental Material 3 (Figure S3.1). Also, we observed a strong and positive correlation (r = 0.93, *P* < 0.001) between the mean SE onboard the ship (Pre-Field) and in the field (Field-1 to -4) (Figure S3.2A). However, there was no significant correlation between Pre-Field SE and the reduction in SE caused by camping (calculated as Pre-Field minus Camp) (r = 0.06, *P* = 0.88). Individual variations in SE can be visualized in Figure S3.2B.

### Analyses of hormones and biomarkers in saliva

**Figure 4:**
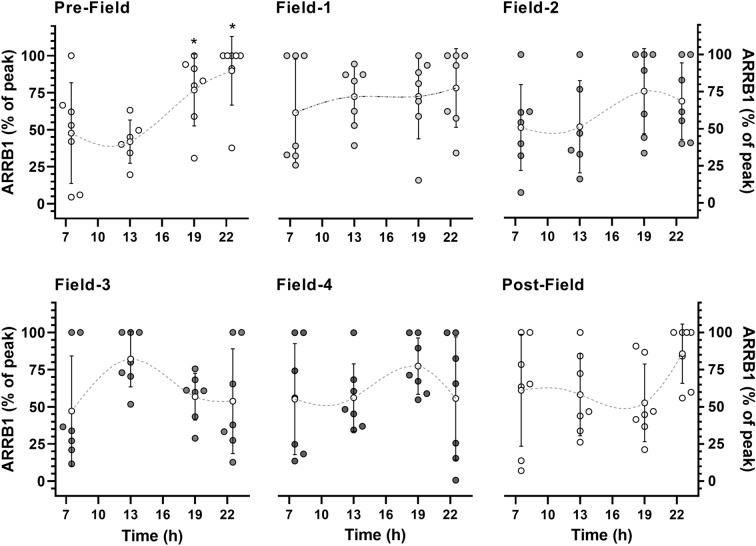
The molecular marker of the peripheral circadian rhythm β-Arrestin 1 (ARRB1), measured in the morning, 30 min after waking up (6:30 h–8:30 h), at 13:00 h, 19:00 h, and 22:30 h, in the following time points: at Pre-Field (i.e., 2nd day on the ship), Field-1, Field-2, Field-3, Field-4 (i.e., 4th, 19th, 33rd, and 45th days in camp, respectively) and Post-Field (i.e., 4th day on the ship). The peak value on each data collection day (for each volunteer) was set to 1 (100%), and the other values of the same day were normalized to the maximum value. The data are expressed as means ± SD. The dots represent individual data. *Significantly different (*P* < 0.05) from morning and 13:00 h. n = 7.

In the Pre-Field, a higher salivary concentration of ARRB1 was observed at night (19:00 h and 22:30 h) compared to daytime (in the morning and at 13:00 h) with a large effect size (F = 5.15, *P* = 0.01; *ES* = 1.7) (Fig. 4). In the camp, this rhythm was no longer observed, with no differences in ARRB1 throughout the day in the Field-1 (F = 0.37, *P* = 0.77; *ES* = 0.4), Field-2 (F = 1.04, *P* = 0.40; *ES* = 0.7), Field-3 (F = 1.64, *P* = 0.21; *ES* = 1.0), and Field-4 (F = 0.66, *P* = 0.58; *ES* = 0.6) measurements (Fig. 3). ANOVA evidenced this rhythm change for acrophase (F = 2.91, *P* = 0.03; *ES* = 1.1) with significant or marginally significant differences between Pre-Field and the other time points (*P-*values from 0.03 to 0.07). In the Post-Field, although ANOVA did not identify differences (F = 2.07, *P* = 0.14; *ES* = 0.3), a large ES suggests a higher value at 22:30 h than the value at 13:00 h (*ES* = 1.1), indicating a return to the Pre-Field pattern. ANOVA did not detect differences in ARRB1 concentration at mesor (F = 1.18, *P* = 0.34; *ES* = 0.8) or concentration amplitude (F = 0.50, *P* = 0.77; *ES* = 0.5).

**Figure 5:**
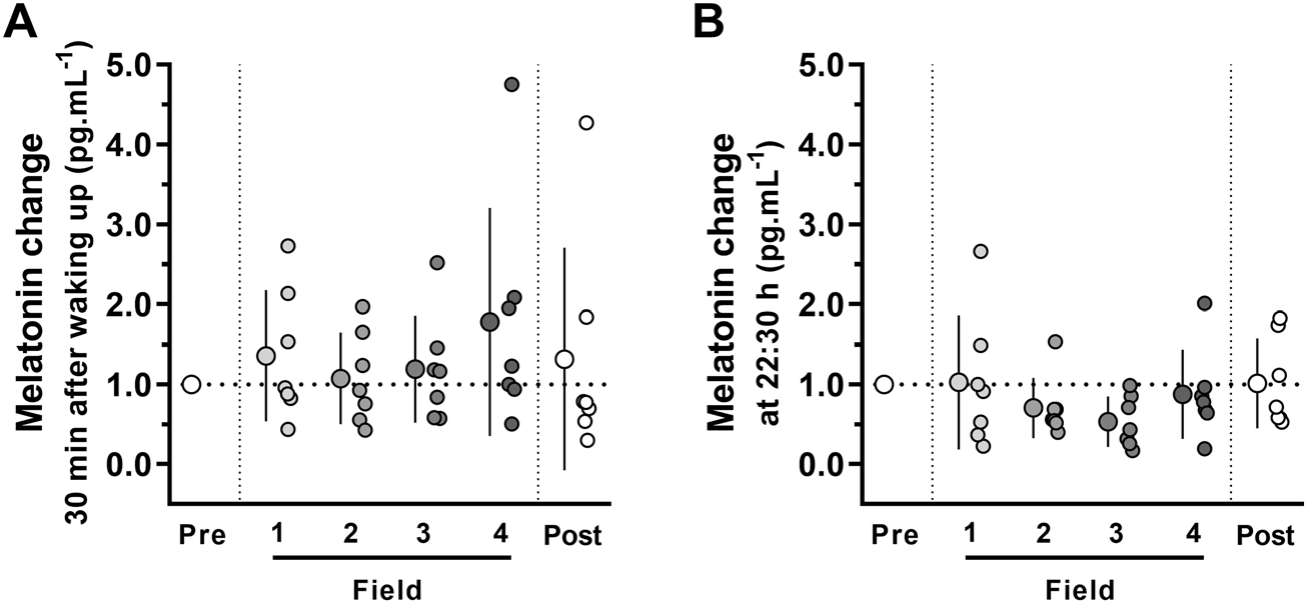
Salivary concentrations of melatonin measured at Pre-Field (i.e., 2nd day on the ship), Field-1, Field-2, Field-3, Field-4 (i.e., 4th, 19th, 33rd, and 45th days in camp, respectively), and Post-Field (i.e., 4th day on the ship): (**A**) melatonin in the morning, 30 min after waking up (6:30 h–8:30 h), (**B**) melatonin at night (22:30 h). Data were normalized to Pre-Field values and are expressed as means ± SD. The dots represent individual data. n = 7, except the Field-1 at 22:30 h, because the measure of one individual was considered an outlier value and excluded.

There was no difference in morning melatonin throughout the expedition (F = 0.62, *P* = 0.68; *ES* = 0.4) (Fig. 5A). For melatonin at 22:30 h, ANOVA showed a tendency for altered concentration over time (*P* = 0.06) (Fig. 5B) with large *ES* from Pre-Field to Field-2 and Field-3 (*ES* = 1.2 and 3.9, respectively). There was a positive and strong correlation between the changes in the nocturnal melatonin concentration and Tsk of the hand (r = 0.958; *P* = 0.002, 6 points), indicating that greater increases in melatonin are associated with increases in Tsk-hand. Furthermore, when considering all time points of the experimental timeline, we observed an inverse significant but weak correlation between nocturnal melatonin concentration and the number of awakenings (r = -0.34; *P* = 0.0275, 41 points), indicating that more melatonin at night is associated with fewer awakenings. However, we should interpret these findings cautiously due to the small sample size and because correlations do not indicate cause and effect.

**Figure 6:**
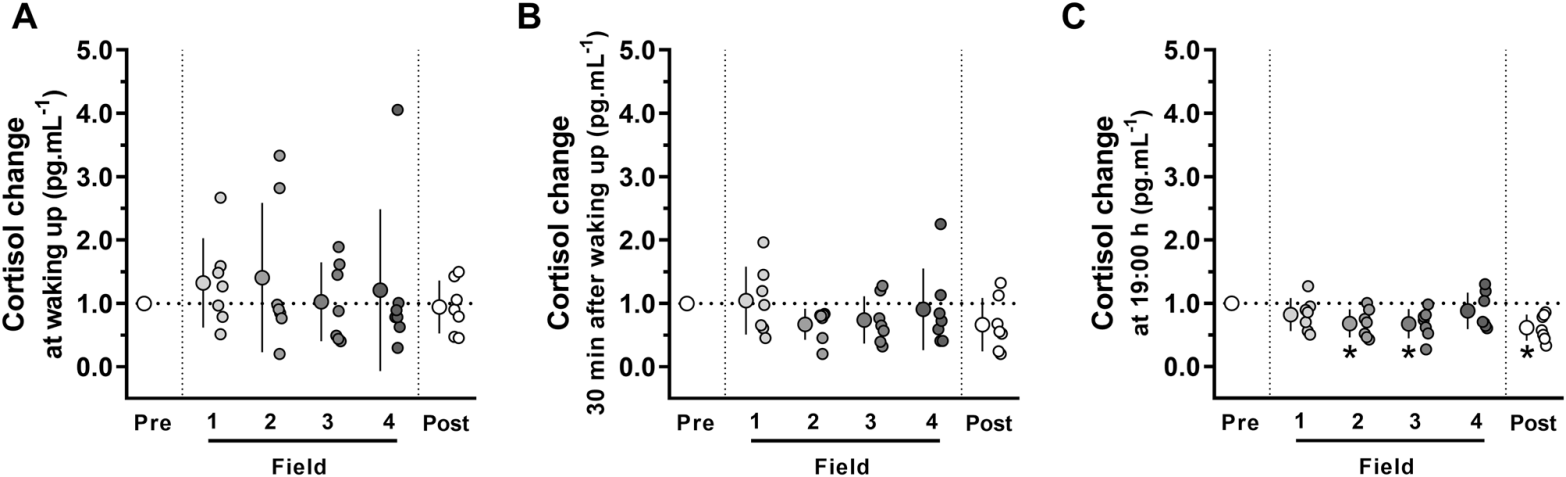
Salivary concentrations of cortisol measured at Pre-Field (i.e., 2nd day on the ship), Field-1, Field-2, Field-3, Field-4 (i.e., 4th, 19th, 33rd, and 45th days in camp, respectively), and Post-Field (i.e., 4th day on the ship): (**A**) cortisol in the morning, at waking up (6:00 h–8:00 h), and (**B**) 30 min after waking up (6:30 h–8:30 h), (**C**) cortisol at night (19:00 h). Data were normalized to Pre-Field values and are expressed as means ± SD. The dots represent individual data. *Significantly different (*P* < 0.05) from the Pre-Field. n = 7.

The expedition did not change the cortisol values measured in the morning, either at wake up (F = 0.47, *P* = 0.80; *ES* = 0.5) (Fig. 6A) or 30 min after waking up (F = 1.92, *P* = 0.13; *ES* = 0.8) (Fig. 6B). However, the expedition moderately influenced cortisol concentration at 19:00 h (F = 4.41, *P* = 0.004; *ES* = 1.1), with lower values observed at Field-2 (*P* = 0.008), Field-3 (*P* = 0.012), and Post-Field (*P* = 0.005) compared to Pre-Field (Fig. 6C). The absolute mean melatonin and cortisol concentrations are presented in Tables S4.1 and S4.2 (Supplemental Material 4).

### Mood responses, daytime sleepiness, and sleep quality

Antarctic camp reduced vigor (F = 5.169, *P* = 0.002; *ES* = 0.7) but did not influence the other mood parameters measured, such as anger, confusion, depression, fatigue, and tension (Table 1). The one-way RM ANOVA revealed that sleepiness, determined through the ESS, tended to increase during camp (F = 2.06, *P* = 0.09; *ES* = 0.6), and its average values indicated excessive sleepiness (scores above 10)^32^ at Field-2, -3, and -4. There were no differences in the subjective sleep index assessed by the PSQI-Total index. However, when the questionnaire domains were considered, the volunteers reported an increase in sleep complaints, like "feel too cold" and "cough or snore loudly" at Field-2, "have pain" at Field-2 and -4, and "cannot breathe comfortably" at Field-2, -3 and -4 (Table 1).

**Table 1:** Mood states, daytime sleepiness, quality of sleep, and sleep complaints during the expedition to Antarctica.

	Pre- Field	Field-1	Field-2	Field-3	Field-4	Post- Field	One-way RM Anova	*ES*
BRUMS mood categories								
Anger	0.8 ± 1.5	1.6 ± 2.6	1.0 ± 1.3	2.3 ± 3.0	2.6 ± 3.1	2.1 ± 3.4	F = 1.22 *P* = 0.32	0.6^M^
Confusion	1.7 ± 2.1	4.1 ± 3.2	1.7 ± 2.2	2.4 ± 1.4	2.3 ± 1.8	2.3 ± 1.2	F = 2.25 *P* = 0.07	0.8^M^
Depression	0.7 ± 0.9	1.6 ± 2.4	1.6 ± 1.9	1.8 ± 2.3	1.8 ± 2.7	2.4 ± 5.1	F = 0.63 *P* = 0.67	0.1
Fatigue	2.4 ± 3.3	3.6 ± 3.9	4.2 ± 2.7	5.1 ± 2.8	4.6 ± 3.4	3.6 ± 4.5	F = 1.57 *P* = 0.19	0.5
Tension	3.3 ± 2.4	5.8 ± 2.7	3.7 ± 1.9	3.1 ± 1.9	4.14 ± 3.23	4.1 ± 3.4	F = 1.58 *P* = 0.20	0.7^M^
Vigor	9.8 ± 3.0	10.0 ± 2.5	7.3*^,1^ ± 4.5	7.6*^,1^ ± 4.3	7.31*^,1^ ± 3.6	7.6*^,1^ ± 3.6	F = 5.17 *P* < 0.01	0.7^M^
Epworth sleepiness scale								
Daytime sleepiness	9.6 ± 3.2	9.9 ± 4.1	12.3 ± 4.9	12.1 ± 5.6	12.0 ± 5.2	9.1 ± 5.4	F = 2.06 *P* = 0.09	0.6^M^
Pittsburgh scale index—*PSQI*								
PSQI-Total index	5.83 ± 2.32	NA	6.71 ± 2.49	5.28 ± 1.80	6.14 ± 2.61	NA	F = 1.05 *P* = 0.39	0.5
Sleep *poor* quality	1.33 ± 0.81	NA	1.14 ± 0.90	0.71* ± 0.48	0.85 ± 0.69	NA	F = 3.54 *P* = 0.04	0.7^M^
Sleep disturbance	1.00 ± 0	NA	1.57* ± 0.53	1.43 ± 0.53	1.43 ± 0.53	NA	F = 3.31 *P* = 0.04	1.0^M^
Sleep latency	1.50 ± 0.84	NA	1.00 ± 0.58	1.00 ± 0	1.28 ± 0.75	NA	F = 1.04 *P* = 0.40	0.7^M^
Sleep *reduced* duration	0.67 ± 0.82	NA	1.00 ± 0.63	0.43 ± 0.53	0.71 ± 0.76	NA	F = 2.05 *P* = 0.15	0.6^M^
Sleep *poor* efficiency	0.67 ± 0.52	NA	0.50 ± 0.84	0.28 ± 0.48	0.43 ± 0.79	NA	F = 0.73 *P* = 0.55	0.5
Use of sleeping medication	0 ± 0	NA	0.14 ± 0.38	0 ± 0	0 ± 0	NA	F = 0.96 *P* = 0.43	0.7^M^
Daytime dysfunction	0.67 ± 0.82	NA	1.57 ± 0.97	1.43 ± 0.79	1.43 ± 0.98	NA	F = 1.89 *P* = 0.16	0.7^M^
Sleep complaints								
Feel too cold	0.66 ± 1.03	NA	2.00^#^ ± 0.82	1.00 ± 0.82	0.71 ± 0.95	NA	F = 4.80 *P* = 0.01	1.3^L^
Feel too hot	0.33 ± 0.82	NA	0.86 ± 1.07	0.28 ± 0.49	0.71 ± 0.95	NA	F = 1.59 *P* = 0.23	0.7^M^
Have pain	0	NA	1.57* ± 1.27	0.57 ± 0.53	0.86* ± 0.90	NA	F = 6.63 *P* < 0.01	1.6^L^
Have to get up to use the bathroom	1.33 ± 1.50	NA	2.43 ± 0.53	2.0 ± 0.82	2.42 ± 0.79	NA	F = 2.37 *P* = 0.11	1.1^M^
Cannot breathe comfortably	0	NA	0.71* ± 0.95	0.86* ± 0.90	0.86* ± 1.21	NA	F = 3.79 *P* = 0.03	0.9^M^
Cough or snore loudly	0	NA	0.71* ± 0.95	0.14 ± 0.38	0.43 ± 0.79	NA	F = 3.23 *P* = 0.05	1.0^M^
Have bad dreams	0.50 ± 0.55	NA	0.86 ± 1.07	0.57 ± 0.79	0.28 ± 0.49	NA	F = 0.90 *P* = 0.46	0.6^M^

Mood state was evaluated using the Brunel Mood Scale (BRUMS), daytime sleepiness was evaluated using the Epworth Sleepiness Scale (ESS), and the quality of sleep and sleep complaints were evaluated using the Pittsburgh scale (n = 7). These data were measured at Pre-Field (i.e., 2nd and 3rd days on the ship), Field-1, Field-2, Field-3, Field-4 (i.e., 4th, 19th, 33rd and 45th days in camp, respectively), and Post-Field (i.e., 4th day on the ship). At Field-2, -3 and -4, excessive daytime sleepiness (score above 10) was observed. The negative dimension was determined by calculating the arithmetic mean of the following dimensions: anger, confusion, depression, fatigue, and tension. *Significantly different from Pre-Expedition. ^1^Significantly different from Field-1. ^#^Significantly different from all other time points. Cohen's d effect sizes (*ES*) were calculated to assess the magnitude of the difference between experimental time points. ^M^Moderate effect size, ^L^Large effect size. The data are expressed as means ± SD. *P* < 0.05. NA: not addressed.

## Discussion

To our knowledge, this research was the first to track the daily sleep pattern during a long summer camp in the Antarctica, showing an impaired sleep pattern characterized by increased fragmentation and waking time after the first sleep episode. Also, transient increases in time in bed and sleep onset latency, associated with reduced sleep efficiency, were detected. We also observed a flattened diurnal difference in salivary concentration of ARRB1 and a tendency to reduce melatonin at night. The volunteers reported physical discomfort due to environmental conditions (feeling of cold and pain, discomfort to breathe, and reports of cough or loud snoring) and presented relatively low proximal-to-peripheral skin temperature during time in bed (the latter, presented in Supplemental Material 2, Figure S2). These psychophysiological changes were accompanied by excessive daytime sleepiness and reduced vigor.

The field period reduced sleep efficiency to levels that may suggest a symptom of insomnia (i.e., below 85%)^40^. The increased WASO and reduced sleep efficiency agree with Pattyn et al.^7^, who carried out polysomnography during one night after three weeks of sleeping in tents in Antarctica. The increase in sleep fragmentation in the present camp also aligns with findings obtained during summer in Antarctica compared to winter, as previously reported by Collet et al.^41^, who investigated twenty-six volunteers living in two research stations: Dumont d' Urville (at sea level altitude) and Concordia (at high-altitude, 3800 m). It is also worth noting that the similar changes were observed in stations at different altitudes, reinforcing the influence of excessive exposure to natural light on sleeping^41^. The data from our research and all the studies mentioned earlier differ from the results presented by Weymouth and Steel^20^, who did not notice differences in sleep assessed in only seven nights. Thus, we suppose that the time interval in their study^20^ was insufficient to detect changes in sleep patterns. Similarly, we did not observe isolated changes in sleep fragmentation, WASO, SOL, and time in bed during the first ten days in the field (i.e., Field-1).

To evaluate if the subjects who had less efficient sleep in Pre-Field also had less efficient sleep in the Antarctic field, we assessed the monotonic relationship between SE data. Spearman's correlation indicated a high and positive association between SE in the field (including values below 85%, a reference to identify insomnia) with SE previously measured on the ship. Thus, the individuals who presented the lowest SE on the ship were the ones who continued to present the lowest values in the field.

Additionally, to evaluate susceptibility to Antarctic field-related environmental stressors and assess whether individuals initially with lower sleep efficiency demonstrated greater deterioration in sleep, we evaluated whether the individuals who presented lower Pre-Field SE were those who presented a greater magnitude of reduction in SE. For this purpose, we analyzed the correlation between pre-Antarctic SE (on the ship, Pre-Field) and the reduction in SE caused by the camp (calculated as Pre-Field minus Camp). However, the lack of a significant correlation between Pre-Field SE and the reduction in SE caused by camping suggests that individuals with less efficient sleep in the pre-Field were not more susceptible to the Antarctic field-related environmental stressors.

SOL presented a biphasic pattern in the field. In December, when the incidence of light at 'night' was higher (sunset between 22:41 h to 22:25 h, from Field-1 to Field 3), we observed an SOL increase. At the end of January, with the anticipation of sunset (between 22:23 h to 21:57 h in Field-4), SOL was reduced. Thus, the light incidence appears to determine the time to fall asleep. Pattyn et al.^7^ reported decreased SOL during one night in a summer camp (about 4 min) compared to a control group at a laboratory in Belgium (about 24 min, compatible with values reported by Bruyneel et al.^42^ for individuals at their homes). The authors suggested that reduced SOL in camp indicates sleep deprivation, as sleepiness emerges when the individuals are removed from the illumination^7^. Thus, in our data, sleep deprivation possibly contributed to the reduction in SOL at the end of the field, alongside the sunset anticipation and the consequent lack of the primary stimulus that inhibits sleepiness (i.e., daylight). Also, it is necessary to consider the adaptation and habituation to the conditions in a tent (e.g., cold, wind noise, and terrain) as adjuvants in reducing the time for sleep onset. For example, a media with nocturnal noise caused by wind blowing on a tent at night (recorded at 2:09 h) is available at 10.6084/m9.figshare.23896263.v1.

The impaired sleep pattern contributes to explaining the tendency to increase nocturnal HR at the end of the field period (Supplemental Material 2, Figure S2). Considering that HR reaches its lowest values (baseline values) during sleep, the increase in HR at the end of the field period may have occurred due to sleep interruption (i.e., more fragmented sleep), leading to stress-related responses that affect cardiovascular autonomic regulation^28^.

**Figure 7:**
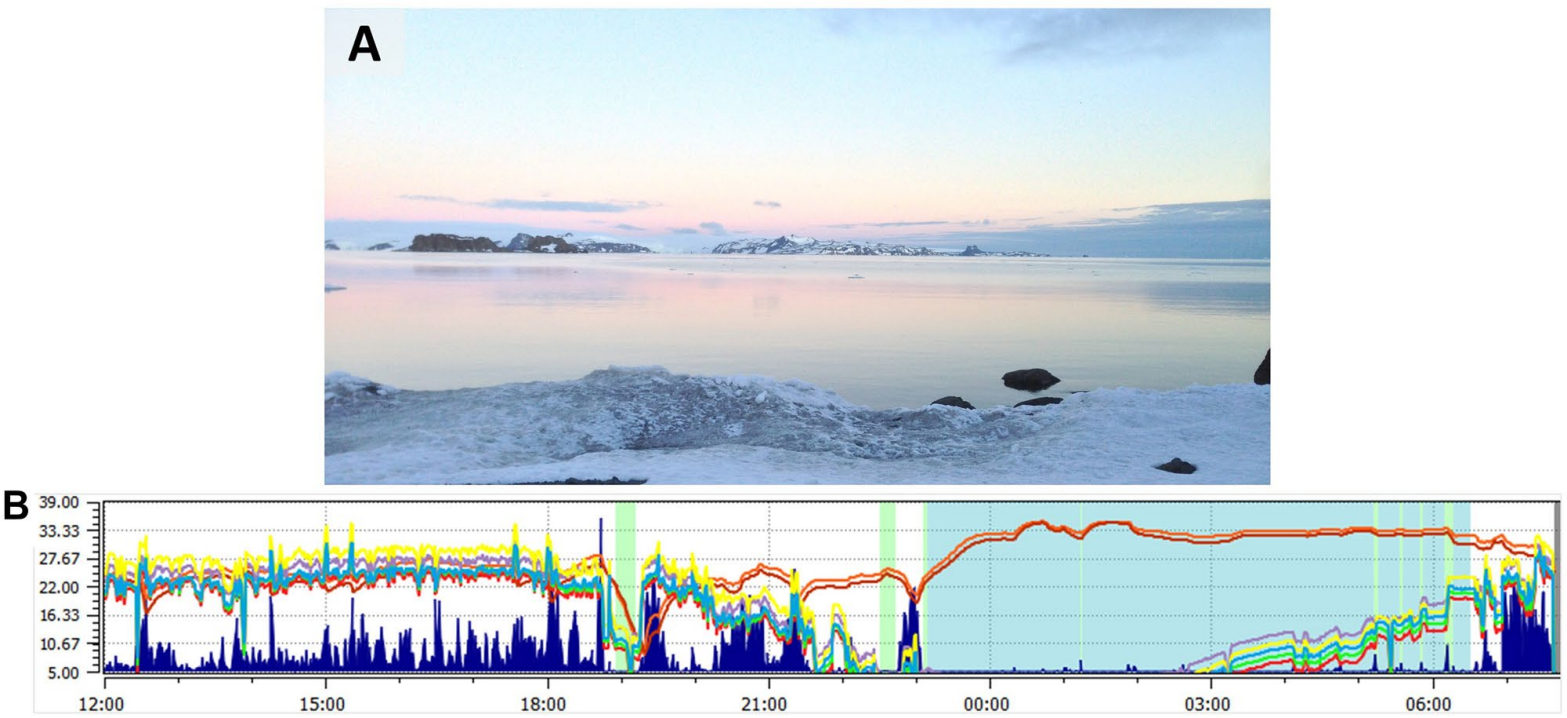
Representation of ambient luminosity. (**A**) View of the campsite recorded at 22:26 h during Field-1 time point. (**B**) Representative recording of luminosity measured overnight by the actigraph (while the participant was inside the individual tent). The blue background marks the sleep period. Maximum luminosity during daytime was 21,883.55 at 15:20 h. A progressive increase of the total visible light (first value above 1 lx; yellow line), infrared light (purple), and red, blue, and green bands can be visualized from 2:50 h. At the end of the sleep period at 6:00 h, the brightness was 56.77 lx. The red lines above the image indicate the wrist skin temperature, simultaneously measured by the actigraph.

Antarctica's ICE conditions, probably the prolonged daily exposure to light (e.g., Fig. 7A), flattened diurnal variation in the salivary concentration of ARRB1. During the permanence in the field, it was no longer possible to observe the diurnal variation seen in the Pre-Field; however, the initial ARRB1 rhythm tended to reappear in the Post-Field. Our data strengthen ARRB1 as a peripheral chrono-biomarker, as proposed by Tomita et al.^16^; however, the time when this biomarker peaked was different from that previously reported: 13:00 h^16^. Differences in light exposure are a possible explanation for this disparity. In the present study, Pre-field data were collected onboard a ship—a closed environment; in the study by Tomita et al.^16^, ambient conditions, especially luminosity, during saliva collection were not described.

Melatonin is a time cue that signals darkness to the body, promoting sleep anticipation and induction^9,43,44^, and possibly its large ES from Pre-Field to Field-2 and -3 contributed to the increased SOL and number of awakenings. Pattyn et al.^7^ reported a melatonin phase-delayed concentration at Princess Elisabeth station. However, a shorter camp in South Shetlands did not change the nocturnal melatonin concentration^5^. An essential difference between our two studies is the day length. In the study by Moraes et al.^5^, when the camp was in late summer (February), the day length was about 2 h to 4 h shorter than in the present study (camp was in December and January), reinforcing the effect of natural luminosity on nocturnal melatonin secretion.

Melatonin synthesis is suppressed by melanopsin^11^, a light-sensitive protein expressed in retinal ganglion cells that transduces light wavelengths into neural input to SCN^45^. Because retinal ARRB1 deactivates melanopsin^46,47^, the attenuated nocturnal peak of salivary AARB1 in the field could have contributed to the large effect sizes observed in the melatonin concentration changes. It is worth noting that, during the day, the luminosity recorded by the actigraphy was approximately 7.5 times higher in the field than on the ship. The average value for the 5 h with the lower luminosity (L5) during sleep was higher in the field than onboard the ship (Supplemental Material 2, Table S2). Illustratively, Fig. 7B shows a progressive increase in luminosity from 2:50 h (first value above 1 lx) until the end of the sleep period at 6:00 h, when the brightness was 57 lx. The luminosity shown in Fig. 7B was obtained by an actigraph inside a tent throughout the night, demonstrating that this luminosity could pass through the fabric of individual tents and be sensed by our participants during the sleep period. Also, it is worth reporting that using a digital lux meter (MLM-1011, Minipa, Brazil), we measured a luminosity of 101 lx around 23:00 h in a single day recording days in the open field (outside the tent). In this sense, Zeitzer et al.^48^ showed that a single exposure to dim room light of 106 lx generates half of the melatonin response observed for a stimulus that is nearly 100-fold brighter.

Interestingly, no differences were observed in morning cortisol, corroborating with Pattyn et al.^7^ and reinforcing the role of social cues and group schedule in cortisol rhythm^7,49^. Nevertheless, reports of increases (or tendency to increase) in morning cortisol exist in short-term scientific camps^5,50^ and at the beginning of a 60-day camp^3^. The longer duration of the present camp may have reduced the time pressure in the first weeks for carrying out tasks, which possibly explains the most evident differences in cortisol during short-term studies in Antarctic camps. As stated by Harinath et al.^3^, specific physical factors (cold, humidity, high wind, and radiation) or psychological stress in Antarctica may represent different stressful stimuli according to the camp duration, resulting in cortisol differences between camps.

Once luminosity induces cortisol release, the decrease in cortisol at 19:00 h cannot be explained by Antarctic summer light; thus, this decline suggests reduced perceived stress throughout the camp. This result corroborates with the findings by Harinath et al.^3^, in which regular cold exposure in Antarctica (after about 30 days) attenuated sympathetic tone and cortisol concentration, possibly due to a lower need for substrate mobilization for heat production. However, van Dalfsen et al.^51^ reported that excessive daytime sleepiness could blunt cortisol reactivity, and this response is aligned with hypocortisolism caused by augmented fatigue levels. Also, the sensitivity of the hypothalamic–pituitary–adrenal axis may eventually decrease when sleep disturbances are persistent^52^. Thus, the changes in sleep patterns, daytime sleepiness, and physical effort over several weeks in the camp may have reduced the cortisol response. Regarding physical effort, a previous study investigating an Antarctic camp with similar routine and fieldwork, intense physical exertion (i.e., time spent in the 70–80 and 80–90% HRmax zones) represented only 9.9% of the fieldwork period and was not continuous^53^. However, despite this low percentage, the absolute time under high-intensity physical exertion corresponded to 24 min per day^53^. Considering the similar characteristics of this and the previous studies, with long walks, and several displacements in areas with slopes and snow, this physical exertion pattern has possibly been reproduced. It is also necessary to consider that reduced cortisol at Post-Field may have been influenced by the reduced light levels on the ship^5,12,14^.

The pattern of mean body and hand skin temperatures along a day was maintained in the camp, with increased skin temperatures overnight (Supplemental Material 2). Individuals preserved their Tsk oscillating from 31 to 35 °C^54^; however, along the nights, the temperatures in the thigh and arm, distal body sites, were lower than in the chest, a proximal body site. During sleep, an approximation of the temperature values in distal and proximal regions is expected^17,54,55^, and a vasoconstrictor tone resulting from the external temperature could reduce sleep efficiency and increase SOL^17,54,56^. The latter observation aligns with our volunteers reporting 'feeling cold' during sleep. In the same direction, we observed a delayed M10 for wrist skin temperature in the field compared to Pre-Field values, reinforcing the role of Tsk on sleep impairment. Unlike thigh and arm skin temperatures, hand skin was not different from the chest temperature, possibly due to behavioral thermoregulation (once while sleeping, individuals usually place their hands close to the body's central region, including the chest and head).

Nighttime Tsk were lower at the end of the field, suggesting insulative acclimatization. This thermoregulatory adaptation reduces skin thermal conductance, boosting body heat retention^57^. Thus, cold acclimatization to Antarctic conditions^3^ may have contributed to the reduced sleep latency observed in the last days of the field. After an initial reduction, the restatement of M10 for wrist temperature is aligned with insulative acclimatization. Also, at the end of the camp, the individuals reported lower scores for 'feeling cold' as a sleep complaint. During camp, we registered the minimum temperature for 42 nights in just one of the tents, where no volunteer slept (range: -3.8 °C to + 3.6 °C) (Supplemental Material 2, Table S2). There was no control over the type of clothing used, and the participants either slept wearing one or two layers of clothing. The lack of clothing control may have interfered with individual thermal comfort, reflecting in the items 'feel too cold' and 'feel too hot'. We suggest that future studies should continuously record the temperatures of all individual sleep environments (i.e., tents) using dataloggers to address the hypothesis concerning insulative acclimatization.

A typical sleep complaint of the volunteers was being unable to breathe properly, which may be the so-called "air hunger" because of the carbon dioxide build-up that occurs when individuals cover their heads with a sleeping bag for warmth^58^. The "air hunger" effect may have contributed to the measured sleep disturbance; however, our present data does not allow confirmation or rejection of this effect. The pain was another sleep complaint declared by the expeditioners and may have resulted from physical demand during the working days. There is an association between sleep and pain once sleep disturbance can produce a hyperalgesic effect and be a stronger predictor of pain^59,60^. In addition, a single night of partial sleep deprivation results in next-day pain reports^59^.

Sleep restriction increases sleepiness and changes individuals' mood under strict routines^61^. Nonetheless, as previously discussed, working in the Antarctic field consists of moderate-to-high intensity physical demands^53^, as shown by the increased activity due to maintaining the camp structure and long hikes with transportation of heavy materials (supplies and samples collected). Considering the role of sleep in restoring physical capacities^62^, it is plausible to assume that the reduced SE impaired the 'individuals' ability to recover, resulting in excessive daytime sleepiness. Additionally, an increased 'confusion' state may have resulted from reduced SE^61^; in this sense, athletes with poor sleep quality reported higher confusion scores than those with good sleep quality^63^.

Despite the altered sleep pattern, perceived sleep quality did not change during the camp. The fact that the participants' 'vigor' remained elevated during camp suggests that vigor may have precluded them from classifying a night with reduced SE as poor perceived sleep. For this reason, perceived sleep quality may not reflect the actual sleep pattern in Antarctic camps. However, a worsening sleep pattern reduces cognitive capacity and physical performance^62,64^, which might affect expeditioners' working demands during Antarctic field. Therefore, individuals must be encouraged to engage in behaviors and use different strategies to improve sleep quality (e.g., thermal insulators and eye masks) and prevent the deleterious effects of ICE conditions.

As in other studies conducted under the extreme conditions of the Antarctic field^53,65–68^, the low number of volunteers is the main limitation of the current study. Due to the limited sample size combined with the loss of degrees of freedom in ANOVAs, some camp-induced changes were visualized through the *ES* analysis. Therefore, it is essential to carry out additional investigations to assess the sleep-related responses in other camps with different participants. Importantly, this limited sample size was the entire camp population investigated and corresponds to the approximate number of individuals generally found in Brazilian Antarctic camps. Additionally, future studies should investigate the relationship between Pre-field and field SE in detail, evaluating SE in more groups/individuals during preparation for the expedition, onboard the ship, and in the field. Also, the independent effects of each environmental stressor found in the ICE conditions on sleep quality should be investigated under controlled laboratory conditions.

Despite the existing limitations, the current findings show remarkable changes in sleep patterns consistently assessed daily throughout a field expedition in Antarctica, along with changes in the emergent chrono-biomarker ARRB1 and alterations in melatonin concentration (a large but only marginally significant effect).

## Conclusion

A prolonged Antarctic camp impairs sleep pattern, with increased fragmentation and waking time after the first sleep episode, thus reducing sleep efficiency. This impaired sleep pattern can be explained collectively by the neuroendocrine changes (as indicated by the chrono-biomarkers), the physical discomfort induced by environmental conditions (feeling of cold and pain, discomfort to breathe, and reports of cough or loud snoring), and the relatively low proximal-to-peripheral skin temperature. Finally, impaired sleep justifies excessive daytime sleepiness during the camp in the austral summer.

### Supplementary Information

Supplementary Information 1.

Supplementary Information 2.

Supplementary Information 3.

Supplementary Information 4.

## Data Availability

The datasets generated during and/or analysed during the current study are available from the corresponding author on reasonable request.
