# Supplementary material for: Sleep impairment and altered pattern of circadian biomarkers during a long-term Antarctic summer camp
Source: Sci Rep. 2023 Sep 25;13:15959. doi: 10.1038/s41598-023-42910-8 (PMC10519969; doi:10.1038/s41598-023-42910-8)
Supplement: Supplementary file 1 — Supplementary Information 1. [file 41598_2023_42910_MOESM1_ESM.docx]

**Characterization of a typical day on the ship and at camp**

This is supplemental material 1 for the article: ***Sleep impairment and altered pattern of circadian biomarkers during a long-term Antarctic summer camp*** authored by Moraes MM, Marques AL, Borges L, Hatanaka E, Heller D, Núñez-Espinosa C, Gonçalves DAP, Soares DD, Wanner SP, Mendes TT, Arantes RME.

**Box S1.**

| **Characterization of a typical day on the ship and at camp** |
| --- |
| - A typical day **on the ship** was characterized by a low level of physical activity and exposure to low-luminosity and warm environments [1]. The volunteers woke up at 7:00 h and had breakfast, following the ship's routine. Lunch, dinner, and a snack were served, respectively, at 11:30 h – 12:30 h, 18:00 h – 18:30 h, and 21:00 h. Between meals, the volunteers engaged in social and work activities (meetings or using personal notebook computers) and watched television. While on board, they usually stayed in sheltered and warm places. - A typical day **in the Antarctic camp** was characterized by activities demanding physical exertion and exposure to a high incidence of natural light and cold environments [1]. Individuals kept a regular daily schedule, with the main sleep episode occurring between 21:30 h and 9:00 h. The average distance traveled by the individuals, measured in 48 days, corresponded to 4.1 ± 0.8 km (ranging from 0.2 to 16.7 km) per day. The field period was characterized by long days and a few hours of twilight. More specifically, the first day had a length of 19 h 42 min (Sunrise: 2:59 h / Sunset: 22:41 h; rest of the night: civil twilight), whereas the last day had a length of 17 h 18 min (Sunrise: 4:29 h / Sunset: 21:47 h; rest of the night: civil and nautical twilight) [2]. Also, high light intensity was recorded in sporadic measurements during Antarctic fieldwork (up to 177,000 Lux) using a digital lux meter (MLM-1011, Minipa, Brazil). - The camp members were six researchers and one climber, and they all followed the same routine. The climber **followed the routine** of the researchers (i.e., paleontologists) and even helped them to prospect. Camp members shared everyday activities (setting up the camp, cooking food, and cleaning). Thus, the routine was similar: the individuals left the camp to the field and returned to the camp together. Upon returning, they made the dinner (two individuals were responsible for dinner each day). After dinner, participants commonly stayed in the social tent to organize research or camping material and socialize with others. |

1. Moraes MM, Bruzzi RS, Martins YAT, et al. Hormonal, autonomic cardiac and mood states changes during an Antarctic expedition: From ship travel to camping in Snow Island. *Physiol Behav*. 2020;224:113069.
2. Zone-related website Time and Date [Online]. Stavanger, Norway, NO 988 375 713 MVA. 2020. https://www.timeanddate.com/sun/@6622142?month=1&year=2020. Accessed March 26, 2022.
