# Supplementary material for: Sleep impairment and altered pattern of circadian biomarkers during a long-term Antarctic summer camp
Source: Sci Rep. 2023 Sep 25;13:15959. doi: 10.1038/s41598-023-42910-8 (PMC10519969; doi:10.1038/s41598-023-42910-8)
Supplement: Supplementary file 2 — Supplementary Information 2. [file 41598_2023_42910_MOESM2_ESM.docx]

**Overnight measurements of mean body skin temperature (Tsk-body) and hand skin temperature (Tsk-hand)**

This is supplemental material 2 for the article: ***Sleep impairment and altered pattern of circadian biomarkers during a long-term Antarctic summer camp*** authored by Moraes MM, Marques AL, Borges L, Hatanaka E, Heller D, Núñez-Espinosa C, Gonçalves DAP, Soares DD, Wanner SP, Mendes TT, Arantes RME

The activity pattern, wrist temperature, and luminosity parameters were continuously measured using a wristwatch. The 24-hour collection of the heart rate (HR) and skin temperature (Tsk) data was performed on one or two volunteers per day, at the beginning (i.e., Field-1, from 4th to 11th day) and end (i.e., Field-4, from 38th to 45th day) of the camp. The recording was started between 18:00 h and 19:00 h, and the volunteers wore the sensors throughout the night and subsequent day.

***Overnight mean body skin temperature (Tsk-body) and hand skin temperature (Tsk-hand)***

Thermochron probes (TH, Maxim Integrated, San Jose, California, USA) were used to measure body Tsk, attached to four skin sites: upper arm (Tsk-arm), chest (Tsk-chest), thigh (Tsk-thigh), and hand (Tsk-hand). The upper arm, chest, and thigh temperatures were recorded every 10 min, whereas hand temperature was recorded every 2 min. Data were downloaded with the OneWireViewer software (TH, Maxim Integrated). The mean Tsk-body was calculated as follows: 0.43 × T-chest + 0.25 × T-arm + 0.32 × T-thigh [1].

***Overnight HR***

A Polar H10 cardiac transducer (Polar Electro Oy, Kempele, Finland) and Polar Beat software in a mobile phone (Polar Electro Oy) were used for data acquisition. Polar Flow online software (Polar Electro Oy) was used for downloading data.

***Activity pattern, wrist temperature, and luminosity measured by actigraphy***

The parameters were evaluated: M10 was the average value of the 10 h after attaining a variable’s highest value, whereas the M10 onset was the start time of the M10 period. The L5 was the average value of the 5 consecutive hours after attaining a variable’s lowest value, and the L5 onset was the starting time of the L5 period. The relative amplitude (RA) was calculated using the equation: RA=(M10-L5)/(M10+L5) [2].

**Results**

***Overnight skin temperatures of the body (Tsk-body) and hand (Tsk-hand)***

There was a large time-of-day effect for Tsk-body (F*=*15.44, *P<*0.001; *ES=* 2.0) (Figure S2.1A) and Tsk-hand (F*=*23.66, *P<*0.001; *ES=*2.8) (Figure S2.1B), with the two temperatures increasing during sleep compared to daytime. During the sleep period (from 01:00 h to 06:00 h), there was a large difference between temperatures measured at different skin sites (F*=*10.74, *P<*0.001; *ES=*2.0) for both the 1^st^ and 2^nd^ measures (Figure S2.1E). The arm and thigh had lower temperature values than the chest (*P*<0.001 and *P*=0.05, respectively), although this was not the case for the hand temperature (Figure S2.1E). ANOVA showed a significant effect regarding expedition time points; Tsk was reduced in Field-4 (F*=*7.79, *P=*0.038; *ES=*0.5) relative to Field-1 during the sleep period (Figure S2.1E), as evidenced by the lower temperatures of the trunk (chest), limbs (thigh, arm) (Figure S2.1C), and hand (Figure S2.1D).

***Overnight HR***

There was a large time-of-day effect in HR, with reduced values during sleep relative to daytime (F=8.69, P<0.001; ES=1.2) (Figure S2.1F). However, considering only data obtained during the sleep period (from 01:00 h to 06:00 h), a moderate effect was observed towards an increased mean HR between the beginning (Field-1) and end of the field period (Field-4), although no significant difference was shown by ANOVA (F=3.35, P=0.11; ES=0.9).

***Activity pattern and wrist temperature***

The Antarctic field largely increased the participants’ activity level, as observed in M10. Compared to Pre-Field, no changes were observed in the Onset M10 for activity; however, in Post-Field, there was a moderate reduction in the Onset M10. No differences were observed for L5, the Onset L5, and the relative amplitude of the activity between the expedition time points mentioned previously (Table S2.1).

For wrist temperature, the M10 value presented a large reduction in Field-1 and a large delay in all field time points relative to Pre- and Post-Field. L5 showed a large decrease in all field time points compared to Pre- and Post-Field. In addition, the Post-Field L5 was higher than Pre-Field L5. There was no difference in Onset L5 for temperature in the field compared to Pre- and Post-Field. The reduction in L5 largely increased the amplitude of wrist temperature throughout the field compared to Pre- and Post-Field (Table S2.1).

***Luminosity by actigraphy***

There was a large difference in diurnal luminosity between the ship and field measurements. The average value for the 10 h with the highest luminosity recorded by actigraphy was about 7.5 times higher in the field than on the ship; the highest values were observed between 8:00 h and 9:00 h in both conditions (ship Pre-Field: 257 ± 134 lx vs. Field: 1,917 ± 1,205 lx, *P*= 0.009, *ES*= 1.9; ship Pre-Field Onset M10: 8 h 50 min ± 0h 20 min vs. Field Onset M10: 8 h 17 min ± 0 h 11 min, *P*=0.002, *ES*=1.9). The average value for the 5 h with the lower luminosity was also higher in the field (Table S2).

**Table S2.** **Pattern of the locomotor activity, wrist temperature, luminosity during or L5 (low 5 h period), and minimum temperature inside a tent at night.**

|  | Pre- Field | Field-1 | Field-2 | Field-3 | Field-4 | Post- Field | One-way RM ANOVA | *ES* | |
| --- | --- | --- | --- | --- | --- | --- | --- | --- | --- |
| *Activity pattern* | | | | | | | |  |  |
| M10  (AU) | 2,654  ±1,145 | 4,422*  ±308 | 3,296  ±401 | 3,964*  ±592 | 3,816*  ±541 | 2,989  ±624 | F = 7.016  *P* < 0.001 | 2.0^L^ |  |
| Onset M10 (h, decimal) | 8.81  ±0.35 | 8.67  ±0.36 | 8.73  ±0.32 | 8.79  ±0.35 | 8.64  ±0.36 | 8.19^#^  ±0.63 | F = 3.481  *P* = 0.013 | 1.1^M^ |  |
| L5  (AU) | 250  ±199 | 305  ±138 | 310  ±192 | 209  ±128 | 214  ±95 | 226  123 | F = 1.631  *P* = 0.182 | 0.6^M^ |  |
| Onset L5  (h, decimal) | 1.56  ±0.96 | 1.55  ±0.25 | 1.74  ±0.27 | 1.33  ±0.35 | 1.44  ±0.55 | 1.25  ±0.58 | F = 0.737  *P* = 0.602 | 0.6^M^ |  |
| RA  (AU) | 0.85  ±0.12 | 0.87  ±0.25 | 0.83  ±0.09 | 0.90  ±0.07 | 0.89  ±0.05 | 0.87  ±0.04 | F *=* 2.136  *P* = 0.088 | 0.6^M^ |  |
| *Wrist temperature* | | | | | | | | |  |
| M10  (°C) | 31.87  ±1.06 | 30.80*  ±0.68 | 31.29  ±0.80 | 31.32  ±1.12 | 31.54  ±0.67 | 32.26  ±0.51 | F= 3.958  *P*= 0.007 | 1.2^L^ |  |
| Onset M10 (h, decimal) | 22.53  ±0.67 | 23.67*  ±0.19 | 23.79*  ±0.24 | 23.12*^,1,2^  ±0.39 | 23.43*  ±0.34 | 22.16  ±0.44 | F = 18.71  *P* < 0.001 | 3.0^L^ |  |
| L5  (°C) | 25.52  ±2.10 | 18.58*  ±1.32 | 21.07*^,1^  ±2.76 | 20.55*^,1^  ±1.98 | 21.25*^,1^  ±2.06 | 28.14^#^  ±0.46 | F = 39.40  *P* < 0.001 | 3.6^L^ |  |
| Onset L5  (h, decimal) | 12.69  ±2.35 | 10.84  ±0.96 | 11.77  ±1.64 | 11.01  ±1.67 | 10.99  ±0.32 | 9.52^#^  ±2.21 | F = 2.664  *P =* 0.042 | 1.2^L^ |  |
| RA  (AU) | 0.11  ±0.03 | 0.25^#^  ±0.03 | 0.20*  ±0.06 | 0.21*  ±0.04 | 0.20*  ±0.05 | 0.07^#^  ±0.01 | F = 29.94  *P* < 0.001 | 3.2^L^ |  |
| *Lumonosity (L5)* | | | | | | | | |  |
| L5  (lx) | 0.024  ±0.025 | 0.606^#^  ±0.394 | 0.178  ±0.112 | 0.094  ±0.058 | 0.092  ±0.067 | 0.029  ±0.036 | F = 13.555  *P* < 0.001 | 2.4^L^ |  |
| Onset L5  (h, decimal) | 24.74  ±0.67 | 23.70*  ±0.64 | 23.76*  ±0.80 | 23.62*  ±0.58 | 23.48*  ±0.47 | 25.46  ±0.77 | F = 9.724  *P* < 0.001 | 2.2^L^ |  |
| *Minimum temperature inside a tent at night* | | | | | | | | |  |
| Temperature  (°C) |  | -1.35  ±2.04 | 1.55^1^  ±1.06 | 2.16^1^  ±1.10 | 0.28  ±1.68 |  | F = 4.746  *P* = 0.009 | 1.4^L^ |  |

Actigraphy registers of the activity and wrist temperature (n=7) calculated as the mean value of Pre-Field (i.e., from 1^st^ to 7^th^ day on the ship), Field-1, Field-2, Field-3, Field-4 (from 1^st^ to 10^th^, 11^th^ to 20^th^, 21^st^ to 35^th^ and 36^th^ to 50^th^ days in camp, respectively), and Post-Field (from 2^nd^ day to 5^th^ day on the ship). *Significantly different from Pre- and Post-Field. ^1^Significantly different from Field-1.^2^Significantly different from Field-2. ^#^Significantly different from all time points, except the onset L5 for temperature, which is different only from Pre-Field. M10 was calculated as the average value of the 10 h following the highest value of the variable. The M10 onset was considered the start time of the M10 period. The L5 was calculated by the average value of the 5 h following the lowest value of the variable. The L5 onset was considered the starting time of the L5 period. The relative amplitude (RA) *= (M10-L5)/(M10+L5).* Arbitrary unit (AU). Lux (lx). The effect sizes (*ES*) for ANOVAs were calculated to assess the magnitude of differences. ^M^Moderate effect size, ^L^Large effect size. For obtaining the temperature inside a tent, we registered the minimum temperature for 42 nights in just one of the tents, where no volunteer slept. We calculated an average value for minimum temperature corresponding to the Field-1, Field-2, Field-3, and Field-4 time points; 5 to 10 nights were recorded for each period. The data are expressed as means ± SD. *P<* 0.05.


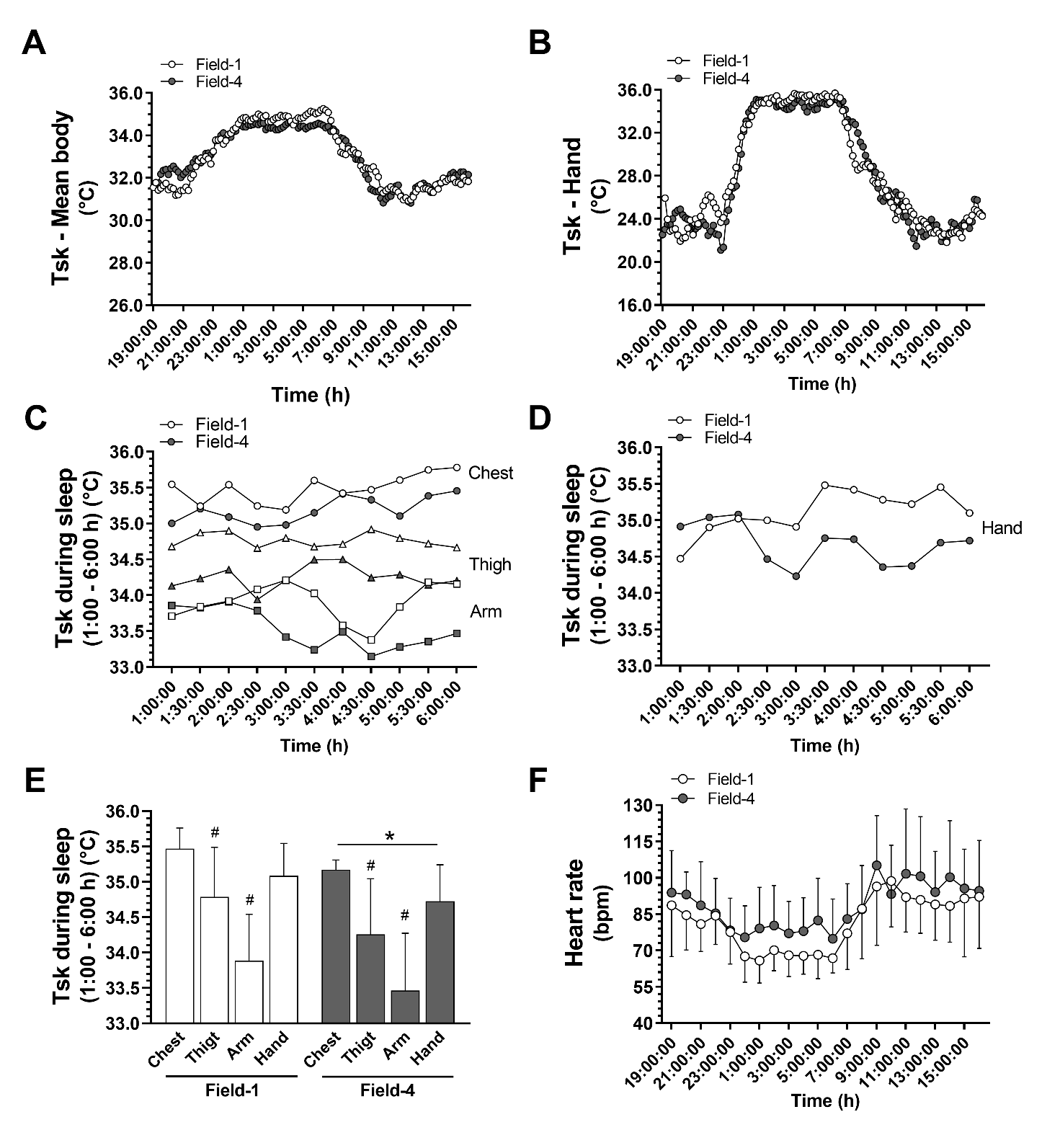


**Figure S2**. **Skin temperatures** **(Tsk) and heart rate (HR) measured over a day at the beginning (Field-1) and end (Field-4) of the camp** (from 4^th^ to 11^th^ and from 38^th^ to 45^th^ days in camp, respectively). A) Tsk-mean body, B) Tsk-hand, C) Tsk measured over the sleep period (1:00 to 6:00 h) in the chest (circles), thigh (triangles), and arm (squares), D) Tsk-hand measured over the sleep period (1:00 to 6:00 h), E) Mean values of Tsk during the sleep period (1:00 to 6:00 h) for chest, arm, thigh, and hand, at the beginning (Field-1) and end of the camp period (Field-4), F) HR over a day and overnight. Beats per minute (bpm). The data are expressed as means ± SD; notably, SD was removed from panels A to D for clarity in data visualization. *Significantly different (*P* < 0.05) from the initial measure. ^#^Significantly different (*P* < 0.05) from chest. n=7.

1. Roberts MF, Wenger CB, Stolwijk JA, Nadel ER. Skin blood flow and sweating changes following exercise training and heat acclimation. J Appl Physiol Respir Environ Exerc Physiol. 1977;43(1):133-137.
2. Van Someren EJ, Lijzenga C, Mirmiran M, Swaab DF. Long-term fitness training improves the circadian rest-activity rhythm in healthy elderly males. J Biol Rhythms. 1997;12(2):146-156.
