# Supplementary material for: Sleep impairment and altered pattern of circadian biomarkers during a long-term Antarctic summer camp
Source: Sci Rep. 2023 Sep 25;13:15959. doi: 10.1038/s41598-023-42910-8 (PMC10519969; doi:10.1038/s41598-023-42910-8)
Supplement: Supplementary file 3 — Supplementary Information 3. [file 41598_2023_42910_MOESM3_ESM.docx]

**The day-to-day data on sleep efficiency and fragmentation during the Antarctic expedition**

This is supplemental material 3 for the article: ***Sleep impairment and altered pattern of circadian biomarkers during a long-term Antarctic summer camp*** authored by Moraes MM, Marques AL, Borges L, Hatanaka E, Heller D, Núñez-Espinosa C, Gonçalves DAP, Soares DD, Wanner SP, Mendes TT, Arantes RME


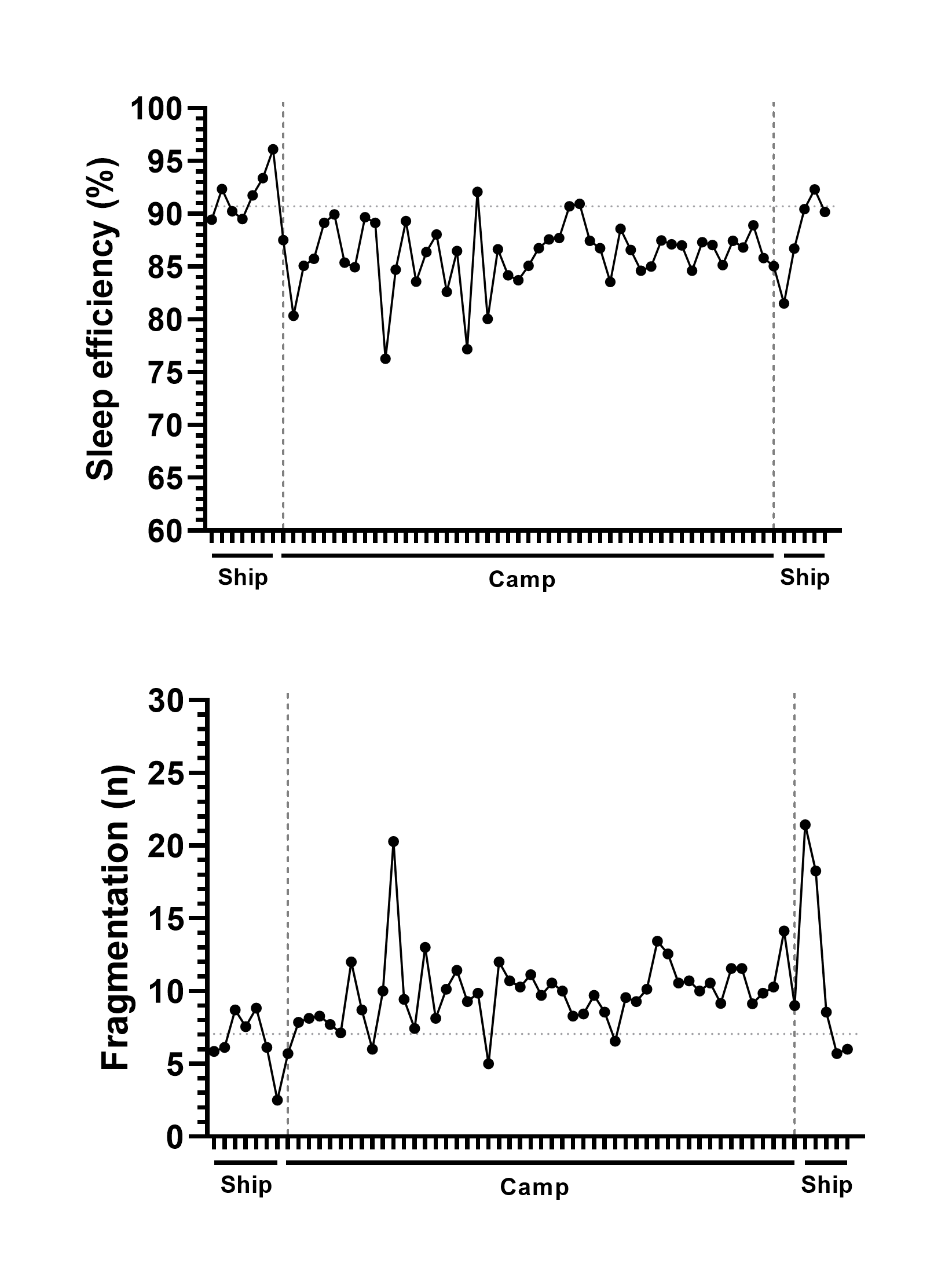


**Figure S3.1.** **Sleep efficiency (above) and fragmentation (below)** at Pre-Field (on the ship), in the field, and at Post-Field (again on the ship). Sleep efficiency, %; the ratio between total sleep time and time in bed. Fragmentation, number; defined as the number of wake events per night. The data are expressed as means. n=7.


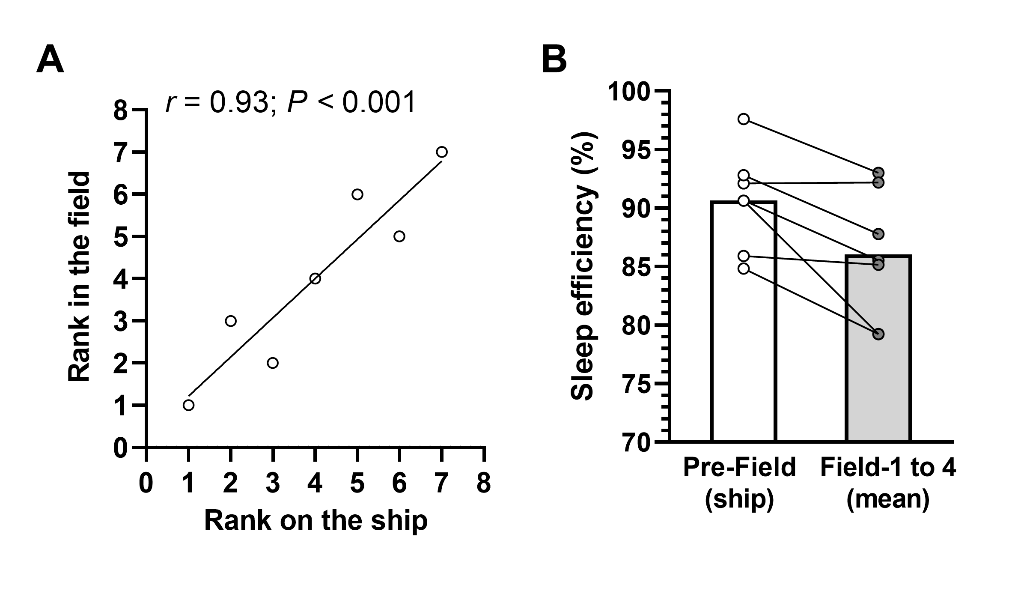


**Figure S3.2.** **Evaluation of sleep efficiency (SE) before the Antarctic camp as a possible predictor of SE in the field.** **(A)** Spearman’s correlation between mean SE onboard the ship (Pre-Field) and in the field (Field-1 to -4). (B) Column graph showing SE at Pre-Field and in the Field. The data are expressed as means, and the lines indicate individual values observed at each moment. SE, %; the ratio between total sleep time and time in bed. n=7.
