# Supplementary material for: Sleep impairment and altered pattern of circadian biomarkers during a long-term Antarctic summer camp
Source: Sci Rep. 2023 Sep 25;13:15959. doi: 10.1038/s41598-023-42910-8 (PMC10519969; doi:10.1038/s41598-023-42910-8)
Supplement: Supplementary file 4 — Supplementary Information 4. [file 41598_2023_42910_MOESM4_ESM.docx]

**Absolute values for salivary melatonin and cortisol concentrations**

This is supplemental material 4 for the article: ***Sleep impairment and altered pattern of circadian biomarkers during a long-term Antarctic summer camp*** authored by Moraes MM, Marques AL, Borges L, Hatanaka E, Heller D, Núñez-Espinosa C, Gonçalves DAP, Soares DD, Wanner SP, Mendes TT, Arantes RME.

**Table S4.1 Absolute values for melatonin measured in the Antarctic camp.**

| Melatonin | Pre- Field | Field-1 | Field-2 | Field-3 | Field-4 | Post- Field |
| --- | --- | --- | --- | --- | --- | --- |
| Melatonin in the morning  at waking up (pg.mL^-1^) | 21.5  ± 16.9 | 22.9  ± 17.0 | 18.2  ± 8.4 | 24.9  ± 25.2 | 29.5  ± 22.7 | 27.9  ± 20.1 |
| Melatonin at night  22:30 (pg.mL^-1^) | 52.4  ± 27.8 | 46.3  ± 47.8 | 32.8  ± 17.7 | 32.9  ± 30.0 | 39.3  ± 24.2 | 41.4  ± 11.2 |

Salivary concentrations of melatonin measured at Pre-Field (i.e., 2^nd^ day on the ship, before field expedition), Field-1, Field-2, Field-3, Field-4 (4th, 19th, 33rd, and 45th days in camp, respectively) and at Post-Field (4th day on the ship). Melatonin in the morning, 30 min after waking up (measured between 6:30 h – 8:30 h). n=7, except for Field-1 at 22:30 p.m., because the measure of one individual was considered an outlier value and excluded.

**Table S4.2 Absolute values for cortisol measured in the Antarctic camp**

| Cortisol | Pre- Field | Field-1 | Field-2 | Field-3 | Field-4 | Post- Field |
| --- | --- | --- | --- | --- | --- | --- |
| Cortisol in the morning  at waking up (pg.mL^-1^) | 1033.2  ± 318.0 | 1221.4  ± 416.3 | 1166.0  ± 651.4 | 968.4  ± 570.3 | 982.8  ± 532.3 | 872.8  ± 253.1 |
| Cortisol in the morning  30 min after waking up (pg.mL^-1^) | 1824.6  ± 1089.6 | 1576.8  ± 665.5 | 1104.5  ± 663.2 | 1130.0  ± 600.0 | 1412.4  ± 998.2 | 1020.1  ± 637.5 |
| Cortisol at night  22:30 (pg.mL^-1^) | 882.6  ± 349.5 | 668.2  ± 184.7 | 552.8  ± 144.5 | 581.9  ± 292.8 | 716.8  ± 169.5 | 521.1  ± 207.6 |

Salivary concentrations of cortisol measured at Pre-Field (i.e., 2^nd^ day on the ship, before field expedition), Field-1, Field-2, Field-3, Field-4 (4th, 19th, 33rd, and 45th days in camp, respectively) and at Post-Field (4th day on the ship). Cortisol in the morning, at wake up (measured between 6:00 h – 8:00 h). Cortisol in the morning, 30 min after waking up (between 6:30 h – 8:30 h). n=7.
